# A New Smart Surface-Enhanced Raman Scattering Sensor Based on pH-Responsive Polyacryloyl Hydrazine Capped Ag Nanoparticles

**DOI:** 10.1186/s11671-017-2257-8

**Published:** 2017-08-14

**Authors:** Shuai Yuan, Fengyan Ge, Man Zhou, Zaisheng Cai, Shanyi Guang

**Affiliations:** 1 College of Chemistry, Chemical Engineering and Biotechnology, Donghua University, 201620 Shanghai, People’s Republic of China; 2 Key Laboratory of Textile Science & Technology, Ministry of Education, Donghua University, Shanghai, People’s Republic of China

**Keywords:** pH-responsive, Ag@PAH NPs, SERS, Ultra-sensitive, Tunable

## Abstract

A novel pH-responsive Ag@polyacryloyl hydrazide (Ag@PAH) nanoparticle for the first time as a surface-enhanced Raman scattering (SERS) substrate was prepared without reducing agent and end-capping reagent. Ag@PAH nanoparticles exhibited an excellent tunable detecting performance in the range from pH = 4 to pH = 9. This is explained that the swelling-shrinking behavior of responsive PAH can control the distance between Ag NPs and the target molecules under external pH stimuli, resulting in the tunable LSPR and further controlled SERS. Furthermore, Ag@PAH nanoparticles possessed an ultra-sensitive detecting ability and the detection limit of Rhodamine 6G reduced to 10^−12^ M. These advantages qualified Ag@PAH NP as a promising smart SERS substrate in the field of trace analysis and sensors.

**Electronic supplementary material:**

The online version of this article (doi:10.1186/s11671-017-2257-8) contains supplementary material, which is available to authorized users.

## Background

Surface-enhanced Raman scattering (SERS) is a powerful spectroscopic tool to identify molecule structure by vibrational information of target molecules [1]. Due to its convenience and ultra-sensitive analysis, SERS has been recognized as an ideal approach to detecting biological molecules, including DNA, RNA and cancer cells [2]. It is generally agreed that SERS technique can be illustrated with the enhanced electromagnetic (EM) [3]. Among the influences of EM, the localized surface plasmon resonance (LSPR) plays a key and dominant role [4]. When target molecules reside in the gaps between neighboring metal nanoparticles (so-called “hot spots”), under the irradiation of incident light, the metal nanoparticle generates LSPR and its surface electromagnetic field is increased, resulting in the enhanced signal of SERS [5–7]. The enormous enhancement ensures the high sensitivity of SERS, which means the characteristic fingerprint of target molecules can be acquired even at low concentrations [8–10].

To date, considerable efforts have been devoted to improve the sensitivity of SERS to develop the technique of SERS analysis. The successful strategies for ultra-sensitive SERS have been realized by metal nanoparticle substrates with different shapes and dimensions [11]. However, to our knowledge, there are no corresponding reports about the controllable SERS detection [12–15]. Therefore, developing tunable SERS will become one of the greatest challenges associated with high sensitivity SERS and biosensors. Polyacryloyl hydrazide (PAH) is a pH-responsive polymer, which has been applied to various biomedical fields [16]. Owing to abundant hydrazide functional groups on PAH, PAH can serve as not only the end-capping reagent but also the reducing agent of the metal ion precursors to easily prepare Ag nanoparticles (NPs) [17]. The swelling-shrinking behavior of responsive PAH can control the distance between Ag NPs and the target molecules under external pH stimuli, resulting in the tunable LSPR and further controlled SERS.

In this work, by combining pH-responsive PAH polymer and Ag NPs, we successfully prepared Ag@PAH NPs without other reagents. Rhodamine 6G (R6G) as the target molecule, Ag@PAH NPs were used to SERS detection for the first time. Due to the responsive of PAH polymer on the surface of Ag NPs, a controllable SERS effect of the R6G/Ag@PAH NPs can be realized by adjusting pH value. Furthermore, Ag@PAH NPs exhibit a high sensitivity and reproducibility, which allow them to be explored for biological hazards or chemical reagent analysis in field applications.

## Methods

**Fig. 1:**
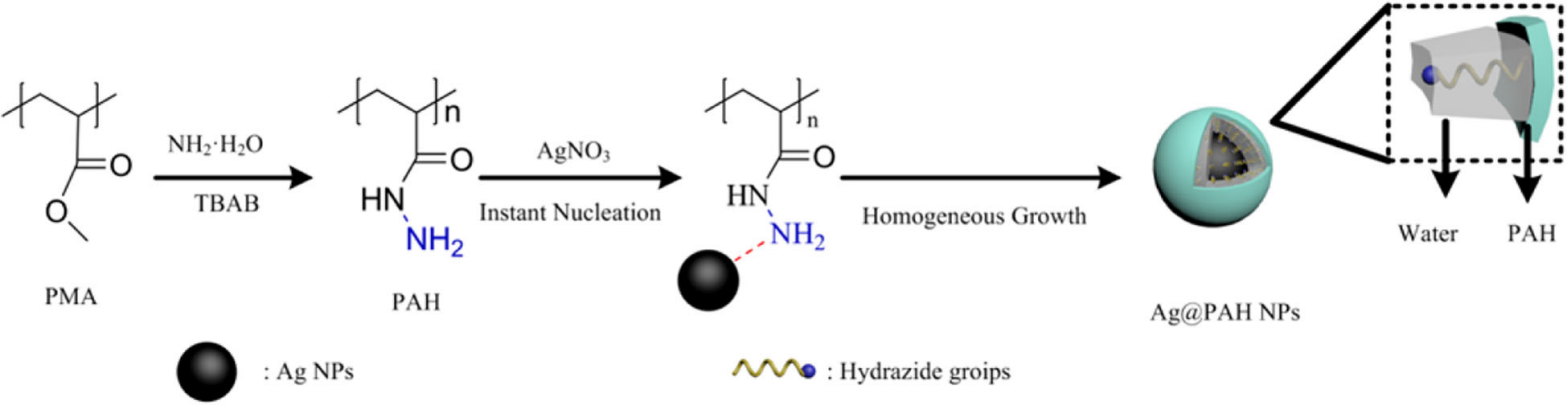
Schematic illustration of the prepared process of Ag@PAH NPs

The illustration of the prepared process of Ag@PAH NPs was shown in Fig. 1. Briefly, 250 μL AgNO_3_ aqueous solution (0.2 mol/L) was added to 25 mL PAH (ESI† for details) aqueous solution (2% *w*/*v*). The mixture was stirred under a mild condition for 30 min at 30 °C. The resulting reddish brown solution was purified by dialysis against deionized water for 24 h and collected by centrifugation and dispersed in deionized water. Then, the different pH values of Ag@PAH NP solutions were adjusted by 0.1 mol/L HCl solution or 0.1 mol/L NaOH solution.

## Results and Discussion

**Fig. 2:**
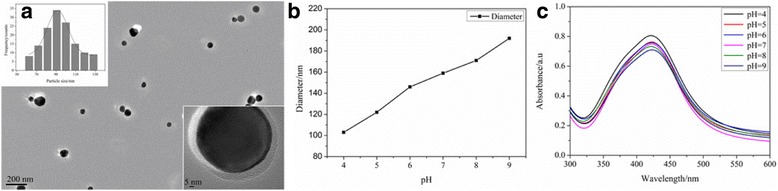
**a** HRTEM images and particle size distribution of Ag@PAH NPs. **b** pH dependence of the hydrodynamic diameter of Ag@PAH NPs. **c** pH dependence of the UV-vis absorption spectra of Ag@PAH NPs

The PAH polymer possessed hydrazide groups in each repeating unit, which served as an effective reducing agent for preparation of metal NPs [18]. Ag^+^ electrophilic substitution, the nitrogen at the end of hydrazide groups, formed -CO-NH-NH- and Ag NPs, in the preparation process of Ag@PAH NPs. By high-resolution transmission electron microscopy, we found that the Ag NPs were fully encapsulated by PAH polymer with the complete core-shell structure. We further estimated that the average size of Ag NPs was about 90 nm in Fig. 2a. The hydrodynamic diameter of the Ag@PAH NPs was 192.6 nm at pH = 9 and decreased to 103.3 nm when the pH value was 4 in Fig. 2b. Moreover, we further calculated the thickness of PAH shell by subtraction of the Ag NP diameter from the total of Ag@PAH NPs which was 102.6 nm at pH = 9 and 13.3 nm at pH = 4. The reason should be attributed to the swell and shrink of the PAH. The swell and shrink of the PAH attributed to a synergistic effect of the following factors, protonation-deprotonation change, charge repulsion, and the hydrogen-bond forming capacity of PAH polymer. In addition, the Ag@PAH NPs showed similar absorption peak (at about 423 nm) in UV-vis spectra and only the absorption intensity decreased in the pH range from 4 to 9 in Fig. 2c. This indicated the increasing thickness of polymer shell layer would hinder the spread of the localized surface plasmon resonance without changing the optical property of Ag NPs.

**Fig. 3:**
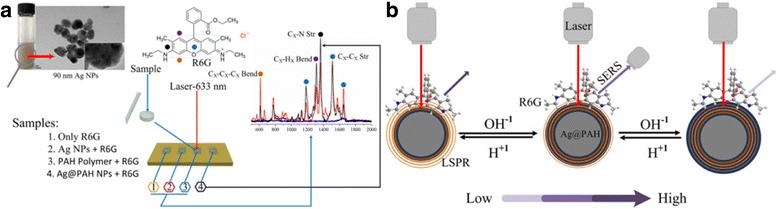
**a** Schematic illustration of the fabrication SERS process of R6G on Ag@PAH NPs substrates. **b** Schematic illustration for the tunable SERS mechanism of R6G with Ag@PAH NPs at different pH values

The SERS performance of Ag@PAH NPs was evaluated with R6G as the model target analyte. In order to understand the origin of the Ag@PAH NPs enhancing R6G Raman signals, compared experiments were performed to distinguish the influence of the PAH polymer layer. We compared the Raman signals of the pure R6G solution, pure PAH solution, individual Ag NPs and Ag@PAH NPs, all of which had the same concentration in Fig. 3a. It is well known that the signal of the pure R6G solution (10^−6^ M) is quite weak. After adding Ag NPs or Ag@PAH NPs as substrates, the main characteristic peaks at 1311, 1363, 1509 and 1651 cm^−1^, which perfectly matched the Ramam spectra of R6G were obviously enhanced. This demonstrates that a remarkable SERS signals from R6G molecules present on the surface of Ag NPs and Ag@PAH NPs. In contrast, in the absence of Ag NPs, negligible SERS signals were observed from individual PAH polymer, suggesting that the presence of PAH polymer had no effect on the SERS effect for R6G molecules.

**Fig. 4:**
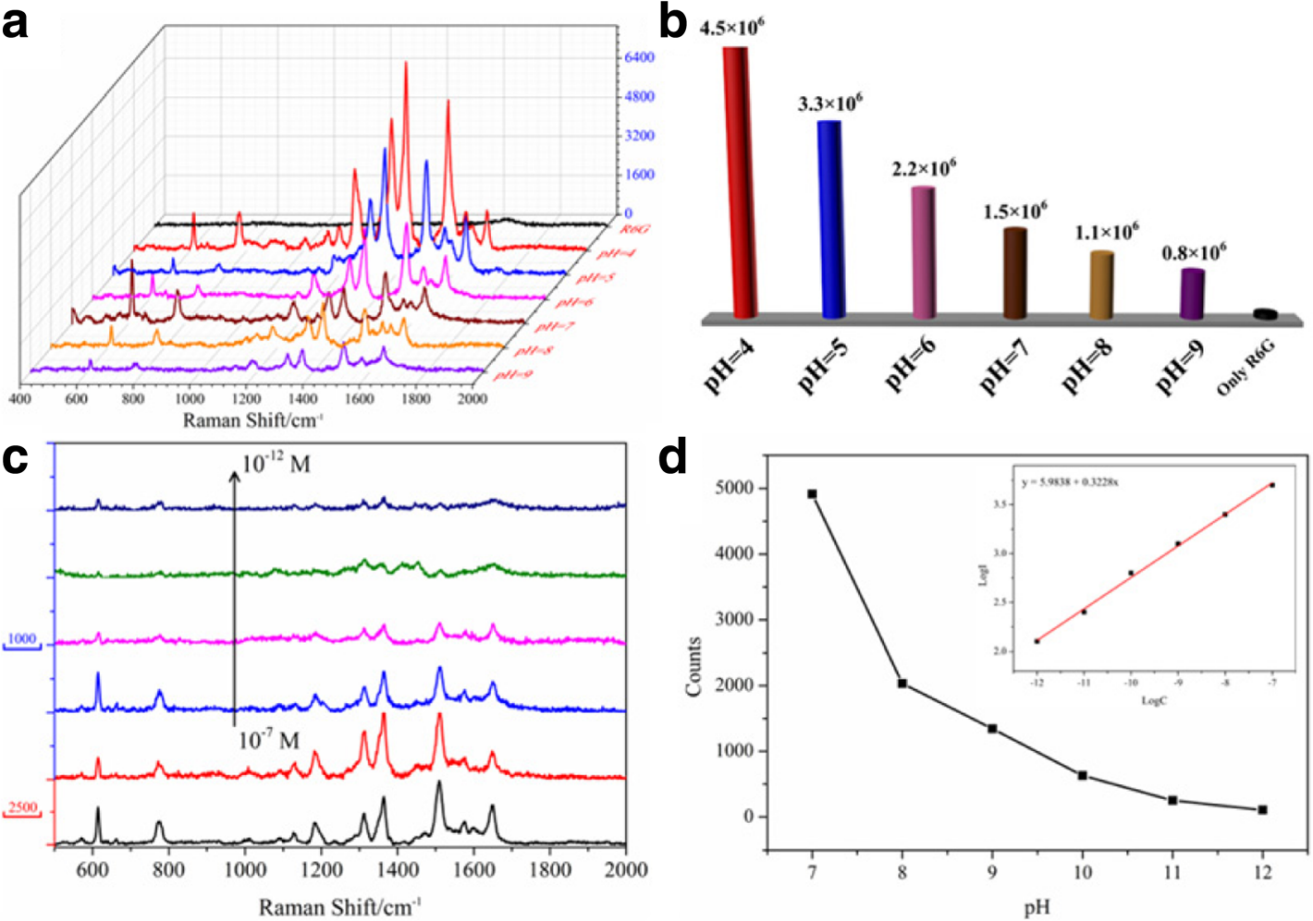
**a** SERS spectra of R6G adsorbed with different pH values. **b** EFs of R6G on Ag@PAH NPs as a function of pH values at 1509 cm^−1^. **c** SERS spectra of R6G with different concentrations adsorbed on Ag@PAH NPs. **d** Relationship of peak intensities at 1509 cm^−1^ and concentrations of R6G (The *inset* is the linear relationship between the logarithmic intensities and concentrations of R6G.)

The SERS enhancement of metal cell/polymer shell was very sensitive to the polymer shell thickness, which has been proved by both theoretical and experimental studies. We investigated the effect between different pH values and SERS-enhanced signals as expressed in Fig. 4a. Compared with the original signal of R6G, the SERS signals were amplified in the presence of Ag@PAH NPs at different pH conditions. Furthermore, the relative SERS intensity of the spectra dropped as the pH value increases. This is explained that SERS effect of Ag@PAH NPs was sensitive to the shell thickness of PAH. PAH shell layer shrank at low pH value, resulting in more intensity of electromagnetic field than that at high pH value in the same concentration of Ag@PAH NPs, as show in Fig. 3b. Therefore, the Ag@PAH NPs at low pH induced extremely enhanced Raman signals, which ensured tunable of the Ag@PAH NPs as SERS substrates. This phenomenon was quantified by calculating the Raman enhancement factors (EFs) of the 1509 cm^−1^ peak for Ag@PAH NPs (Eq. S1, ESI†). The EFs of Ag@PAH NPs at different pH values were estimated to be 0.8 × 10^6^, 1.1 × 10^6^, 1.5 × 10^6^, 2.2 × 10^6^, 3.3 × 10^6^ and 4.3 × 10^6^, respectively, in Fig. 4b (ESI† for details). The EFs of Ag@PAH NPs at different pH values were all high, up to 10^6^ which revealed that the Ag@PAH NP could be used as an effective and intelligent SERS substrate in the trace detection.

In addition, Ag@PAH NPs at low pH value induced extremely enhanced Raman signals, which ensured ultra-sensitivity of the Ag@PAH NPs as SERS substrates. Therefore, a series of SERS spectra of R6G at different concentrations (10^−7^–10^−12^ M) were further measured at pH = 4 with adding Ag@PAH NPs at the same concentration. Comparing the signals of these curves, the SERS intensities were decreased by diluting the concentrations of the target molecule in Fig. 4c. The characteristic bands of R6G are identified clearly even at a concentration as low as 10^−12^ M, demonstrating Ag@PAH NPs possess a high detected sensitivity for R6G. Furthermore, a linear dependence is found between the logarithmic concentrations of R6G and the intensities of the fingerprint peak (1509 cm^−1^) in Fig. 4d. When in the concentration range of R6G ranged from 10^−7^ to 10^−12^ M, the linear regression equation was *y* = 5.9838 + 0.3228 log(x), and the correlation coefficient was 0.9971 (*n* = 6). Obviously, in the low concentration region, SERS intensity decreased with the test concentration decreases. These results confirmed that the Ag@PAH NPs will become a promising candidate in a smart ultra-trace detection of biological hazards or chemical reagents.

## Conclusions

In summary, we utilized pH-responsive Ag@PAH NPs as desired substrates for SERS applications for the first time. The introduction of pH-responsive PAH polymer as a shell layer can endow Ag NPs a controllable localized surface plasmon resonance by adjusting the shell thickness under pH stimuli, resulting in tunable SERS effects. The results demonstrated that Ag@PAH NPs possessed excellent controllable pH-responsive and ultra-sensitive SERS performance which the detection limit of R6G reduced to 10^−12^ M. Ag@PAH NPs are promising for the smart SERS application in the ultra-trace detection of biological hazards or chemical reagents.

## Associated Content

Supporting information. Materials, intstrumentation, preparation of PAH and EF caculation method. Figure S1. ^1^H NMR spectrum of PMA in CDCl_3_ and PAH in D_2_O (Additional file 1).

## Additional file

Additional file 1: Supplementary material. (DOCX 265 kb)

## Abbreviations

EFs: Enhancement factors
EM: Enhanced electromagnetic
LSPR: Localized surface plasmon resonance
NPs: Nanoparticles
PAH: Polyacryloyl hydrazide
SERS: Surface-enhanced Raman scattering
