# Supplementary material for: A New Smart Surface-Enhanced Raman Scattering Sensor Based on pH-Responsive Polyacryloyl Hydrazine Capped Ag Nanoparticles
Source: Nanoscale Res Lett. 2017 Aug 14;12:490. doi: 10.1186/s11671-017-2257-8 (PMC5555959; doi:10.1186/s11671-017-2257-8)
Supplement: Supplementary file 1 — Supplementary material. (DOCX 265 kb) [file 11671_2017_2257_MOESM1_ESM.docx]

**Supplementary material**

for

**A New Smart Surface-Enhanced Raman Scattering Sensor Based on pH-Responsive Polyacryloyl hydrazine capped Ag nanoparticles**

Shuai Yuan ^a^, Fengyan Ge ^a, b^ *, Man Zhou ^a^, Zaisheng Cai ^a^, and Shanyi Guang ^a^

^a.^ *College of Chemistry, Chemical Engineering and Biotechnology, Donghua University, Shanghai 201620, People's Republic of China. E-mail: fyge@dhu.edu.com; Tel: +86-21-67792608-804.*

^b.^ *Key Laboratory of Textile Science & Technology (Donghua University), Ministry of Education.*

**Experimental**

**Materials**

Rhodamine 6G (R6G) and tetrabutyl ammonium bromide (TBAB) were purchased from Sigma Aldrich and used without further purification. Other chemicals and solvents were purchased from Shanghai Chemical Reagent Co. and used without further purification. All deionized water was used in the process of synthesis and characterization.

**Instrumentation**

^1^H NMR data were recorded on a Bruker AV400 spectrometer. Transmission electron microscopy (TEM) images of the Ag@PAH NPs were observed using a 2100F transmission electron microscope (JOEL, Japan) at a voltage of 200 kV and the diluted Ag@PAH NPs dispersions were dropped onto carbon coated copper grids and dried before observation. The hydrodynamic diameters (Dh) of the Ag@PAH NPs dispersed in the various pH buffer solution were detected via BI-200SM dynamic light scattering (DLS) with BI-9000 AT digital time correlator (Brookheaven, USA) in the condition of 90^o^ for scattering angle. Laser source is a He-Ne laser with 633 nm and 35 mV. The UV-visible spectra of the Ag@PAH NPs dispersed in the waters of various pH values were tested via a UV-visible spectrometer (PerkinElmer, USA). The Raman spectra of the Ag@PAH NPs were measured on a in Via-Reflex micro-Raman spectroscopy system (Renishaw, UK) with a 633nm solid laser source of 50 mW power.

**Preparation of PAH** PAH was synthesized using a procedure reported earlier. Polymethyl acrylate (PMA) were prepared according to Mahaveer’s report.[^1^](#_ENREF_1) Then PMA (0.156 mol) were dissolved in 300 mL tetrahydrofuran, hydrazine hydrate (0.8 mol) and TBAB (0.031 mol) were added to it. The mixed solution was heated to 60 °C and lastly reacted for 12 h. The prepared PAH solution was transferred into methanol to form the polymer product. The obtained white product was washed several times and dried at ambient temperature under reduced pressure for further use.


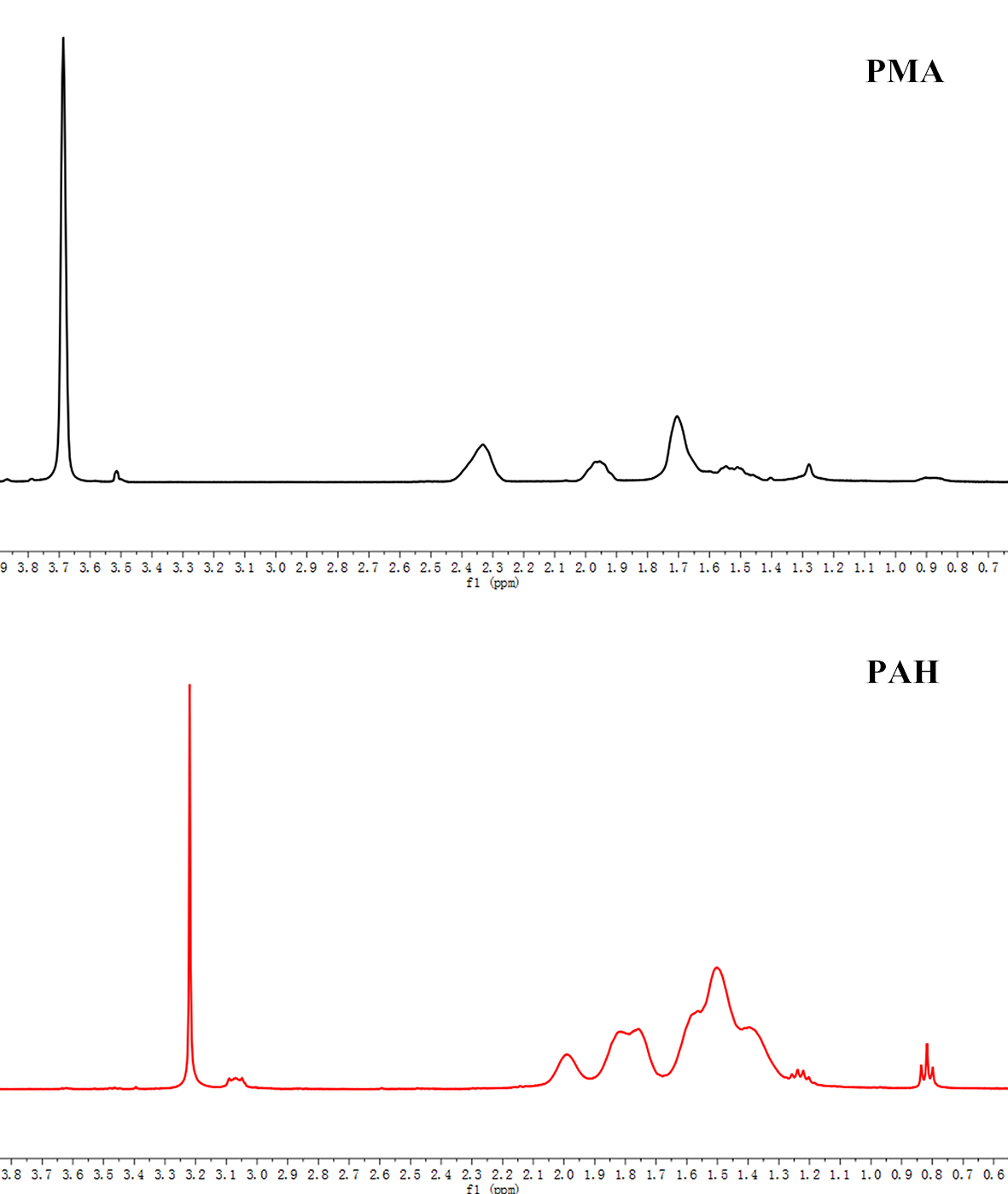


**Fig. S1** ^1^H NMR spectrum of PMA in CDCl_3_ and PAH in D_2_O

**SERS enhancement factor (EF) calculation**

The EF is an important parameter for the performance of SERS substrates.[^2^](#_ENREF_2) The same method is applied to calculate EFs for other samples. The 1512 cm^-1^ peak of R6G was chosen to calculate the SERS EF. The SERS EF is calculated from the standard equation defined as:

 equation S1

Where I_SERS_ and I_Raman_ are the Raman intensities of the 1512 cm^-1^ peak for R6G adsorbed on Ag@PAH NPs solution and a pure R6G solution. N_SERS_ and N_Raman_ are the numbers of the adsorbed molecules on Ag@PAH NPs and 10^−1^M R6G solutions without adding Ag@PAH NPs. In detail, for determination of N_SERS_ and N_Raman_, due to the same magnification (× 50) and other experimental conditions, such as the exposure time and the power of laser, are identical in all cases. The concentrations of for SERS and unenhanced Raman analyses were 10^−6^ and 10^−1^, respectively. Therefore, we can know the ratio N_Raman_ / N_SERS_ was about 10^6^.

Notes and references

1. S. Yuan, F. Ge, Y. Chen and Z. Cai, *RSC Advances*, 2017, **7**, 6358-6363.

2. T. Y. Jeon, S. G. Park, S. Y. Lee, H. C. Jeon and S. M. Yang, *Acs Applied Materials & Interfaces*, 2013, **5**, 243-248.
